# Study on eye movement characteristics and intervention of basketball shooting skill

**DOI:** 10.7717/peerj.14301

**Published:** 2022-10-31

**Authors:** Qifeng Gou, Sunnan Li, Runping Wang

**Affiliations:** 1 College of P. E and Sports, Beijing Normal University, Beijing, China; 2 College of Physical Education, The Northwest Normal University, Lanzhou, Gan Su, China

**Keywords:** Basketball, Shooting, Eye movement, Aiming point, Intervene

## Abstract

**Background:**

The shooting aiming point is very important in basketball because it may affect the field goal percentage (FG%). The purpose of this study was to explore the influence of shooting aiming point practice on FG% and to search for new training methods for shooting improvement in basketball.

**Methods:**

A total of 24 expert basketball players and 24 amateur basketball players participated in the shooting task of Experiment 1. The participants in the two groups wore an eye movement instrument while shooting the ball. The shooting techniques included free throws, 45° direct shots and 45° bank shots to verify the differences in shooting aiming points between expert basketball players and amateur basketball players. Forty-eight amateur basketball players participated in the teaching experiment of Experiment 2. Twenty-four participants participated in routine teaching, and 24 participants had shooting aiming point practice for nine weeks to verify the difference in FG% between the two groups. The shooting aiming points of the participants were assessed immediately after shooting.

**Results:**

Experiment 1 found that expert basketball players used shorter fixation duration, fewer fixation numbers and more reasonable (simple and efficient) fixation distributions than amateur players. Moreover, expert basketball players took the front edge of the hoop as the aiming point, and amateur players took the central or back edge of the hoop as the aiming point; the FG% of the expert group (83.47%) was significantly higher than that of the amateur group (34.86%) (*P* < 0.01). Experiment 2 found that for the total FG% of the three tests, the intervention group (30.19%) was significantly higher than that of the control group (27.27%) (*P* < 0.05). After five weeks of aiming point training, it can be found that was no significant difference in the FG% between the intervention group (28.19%) and the control group (26.53%) (*P* > 0.05). After 9 weeks of shooting aiming point training, the FG% of the intervention group (36.39%) was significantly higher than that of the control group (30.14%) (*P* < 0.05), and the FG% of the intervention group increased faster than that of the control group. Additionally, the aiming point of the intervention players changed from the center and back edge of the hoop to the front.

**Conclusion:**

(1) There was a correlation between basketball shooting aiming point and FG%. FG% with the front edge of the hoop as the aiming point was higher than the back edge hoop or center. (2) The FG% could be more quickly improved by shooting aiming point practice; it will not be affected in a short time (5 weeks); however, 9 weeks of practice can significantly improve the FG%.

## Introduction

Shooting quality is mainly determined by the mechanics of shooting at the basket, the positions of the head and eyes (due to the fact that looking at the target has an important role in improving shooting precision), and concentration (due to the fact that it is an important role in optimizing shooting precision) (Netolitzchi et al., 2019). Attention is considered to be one of the most vital factors for motor learning and performance (Wulf & Prinz, 2001). The especially necessary factor for self-paced (Singer, 2010) and closed-skill tasks is focused attention (Loze & D Collins, 2001), such as the precision sports of basketball free throws. Attention is seen as the control of the detection of information (Moeinirad et al., 2020), and the focus of external attention affects optimal performance (Lewthwaite & Wulf, 2017; Wulf & Lewthwaite, 2016). Shooting is a complex aiming skill that requires the combination of visual information obtained through precise visual aiming and effector actions for performing aiming actions (França et al., 2021). Shooting techniques need to pay attention to the narrow range and point to the internal information, and can sensitively grasp various physical feelings and accurately diagnose technical and tactical problems (Nideffer, 1976). Experienced players detected critical visual information likely to predict shot success by properly moving their gaze according to the shooter’s movements (Mizuguchi, Honda & Kanosue, 2013). When vision was occluded just before initiating the shooting movement, there were marked decrements in performance. In basketball shooting, the two main shots include the direct shot (as is most often used with the free throw) or the bank shot (as a shot made after rebounding off the backboard), and the two major shooting styles may be distinguished (Oudejans, Langenberg & Hutter, 2002). The field goal percentage is a common and efficient measure of shooting skill, which is the number of made shots out of the total number of shots (Daly-Grafstein & Bornn, 2019).

It has been previously demonstrated that jump shooting can be successfully performed while viewing the basket for just 397 millisecond (ms) (Oudejans, Langenberg & Hutter, 2002). Furthermore, researchers using an intermittent viewing technique found that picking up visual information at later time points in both low- and high-style shooters characterized the expert performance of the jump shot (Oliveira, Oudejans & Beek, 2006). They found that basketball jump shot performance was deterred when visual information was unavailable during movement execution (Oliveira et al., 2007). Collectively, the findings that underscored the importance of the online use of visual information in basketball shooting (Oliveira, Oudejans & Beek, 2008).

Field goal percentage is a common measure of shooting skill and efficiency in the International Basketball Federation (FIBA), and general shooting prowess is often defined for players by their overall FG% (Daly-Grafstein & Bornn, 2019). The shooting percentage is closely related to many factors, and the selection of the aiming point is crucial to this percentage (Chen & Ren, 2006). The movements of the eyes and head are usually smooth; once the attention is stabilized on the target, the information is processed (Quintana et al., 2007). Therefore, attention behavior in shooting is defined as how an individual turns his head and eyes during preparation and shooting to obtain available information (Wang, Wang & Yang, 2008). The information is directly aimed at the target to ensure the accurate completion of the action. Moreover, the eye muscles which need to input information to cause the command proprioception at the level of the neck muscles is thought to play a role as they provide angle of elevation information for the shot (Galotti, 2016; Oliveira, Oudejans & Beek, 2009).

It is essential for the shooter to attend to the appropriate information visually, which for releasing an accurate shot (Kim et al., 2021). Previous studies have indicated that experts efficiently attend to information which relevant for their actions while leaving irrelevant and potentially distracting information unattended (Milton et al., 2007). Pistol expert players will first aim at the target during target shooting, whereas novice players will first aim the gun and then focus on the target (Ripoll et al., 1985). Compared with novice players, expert players can aim at the hoop faster and maintain attention for a longer period of time (Kanat & Simsek, 2021; Rienhoff et al., 2013), and the quiet eye during shooting can fulfill an online control function (Giancamilli et al., 2022). There have been inconsistent conclusions about the position of the shooting aiming point (Chen & Ren, 2006; Mu & Li, 2015). In previous studies, due to instrument limitations, helmet-mounted eye trackers have been shown to interfere with players’ shooting percentages and easily cause the visual fatigue of players, which can affect the experimental results (Yan & Bai, 2018). Using a wearable eye tracker can effectively solve this problem. However, there has been little research on shooting skill combined with aiming point practice. Moreover, the question of the duration of practice needed to improve FG% needs to be effectively explored.

This study was designed and based on the shooting aiming point on FG%. Experiment one used a wearable eye tracker to explore the eye movement characteristics of expert and amateur basketball players when shooting. We assumed that there are different aiming points between the expert group and the amateur group in free throws, direct shots and bank shots, which can affect the difference in FG%. Experiment two involved the intervention of amateur basketball players’ shooting aiming points. We assumed that shooting aiming point practice could improve FG%.

## Materials & Methods

### Participants

The sample size was estimated using the software G*Power3.1.9.7 (Germany) (Faul et al., 2007). When considering an effect size of 0.90 based on similar studies (Qiu et al., 2019), an alpha level of 0.05, a power of 0.80 and two tails (Cohen, 1992), a sample size of 21 individuals per group was determined. To allow for dropouts, we selected 24 participants per group, with a total of 48 participants. In Experiment 1, a total of 24 expert players selected from the Northeast Division of the China University Basketball League Division One (Jin et al., 2020), with more than 10 years of experience per person (mean: 10.86 years; *SD*: 2.02 years) and then their ages ranging from 19 to 23 years (mean: 21.51; *SD*: 2.31 years), comprised the basketball player group. All of the players participated in the experiment. The amateur group was composed of 24 undergraduate students aged 18–22 years (mean: 19.57; *SD*: 1.52 years) with no basketball training experience. In Experiment 2, 48 participants (24 participants per group) were from the sophomore basketball optional course. The intervention and control groups were composed of 48 undergraduate students between the ages of 19 to 21 years (mean: 20.45; *SD*: 0.96 years) with no basketball training experience, except for routine teaching. All participants were right-handed and reported having a normal or corrected-to-normal vision. Each participant who completed the study was compensated for their time. The experimental protocol was approved by the regional ethics committee of Northwest Normal University (No. 20210812). All of the participants provided written informed consent prior to the start of the experiment.

### Design

Experiment 1 consisted of a shooting experiment for basketball experts and amateur players. We used a 2 (groups) ×3 (shooting techniques) ×3 (area of interest, AOIs) experimental design, group (expert group and amateur group) as the interparticipant variable and shooting technique (free throw, 45° direct shot and 45° bank shot) as a variable within the participant. Shooting percentage and eye movement indicators were the dependent variables, AOIs were hoops, boards and nets. Shooting skill is measured by field goal percentage typically, which is the number of shots made out of the total number of shots. Experiment 2 included the intervention experiment of the shooting aiming point in basketball teaching. An experimental design of 2 (groups) ×3 (shooting techniques) ×3 (times) was adopted, with groups (intervention group and control group) as the interparticipant variable, shooting technique (free throw, 45° direct shot and 45° bank shot; bank shot as a shot made after rebounding off the backboard) as a variable within the participant and times (before, during and after the intervention) as the intraparticipant variable. Moreover, the shooting percentage was the dependent variable.

In contrast, the control group performed routine teaching. The training content is basketball technology teaching, which carried out according to the routine teaching content and schedule of the school. On the basis of the control group, the intervention group had 15 min of aiming point training in class. The PE teacher focused on explaining the position of the aiming point before each participant shot and on reminding participants to pay attention to the fixation location when they shot. To ensure the enthusiasm of the participants and the quality of the experiment, the participants were told not to participate in extra basketball practice during the experiment. The experiment was accompanied by 2 testers to ensure its completion.

All participants shot in the same position and performed a standing shot. A standard basketball court was used for the basketball free throw task. The rim was 3.05 m high, and the free throw line was at a distance of 4.23 m to the middle of the hoop, which are both standard measures according to international basketball rules by the International Basketball Federation (FIBA) (Rienhoff et al., 2013). The position of the 45° direct shot and bank shot was at a perpendicular distance of 5 m from the basket, and the angle with the bottom line on the right side was 45°. The shooter’s position was marked on the floor with white tape to ensure that the shooting position is exactly the same in each test. After giving instructions, the shooter practiced shooting several times to warm up and adapt to the equipment. When the shooter said that he had warmed up, the experiment began to test. Each shooter was instructed to take ten free throws, 45° direct shots and 45° bank shots in turn, without the time limit, which would be tested by four same experimenters. After ten shots, there was a short break of 1–2 min. Immediately after completing the test, all of the shooters were interviewed as to whether there was a shooting aiming point, where the aiming point was and whether there was any change in the shooting aiming point before and after the intervention experiment.

### Apparatus

Tobii Pro Glasses3 (Sweden; 106° H: 95° , V: 62°) is a wearable eye tracker that exerts no obstructions to the wearer’s field of vision and provides maximum freedom of head and body movements without affecting the data quality. We ensured that natural and real behavior was captured to the greatest extent. The sampling rate of 50 Hz can be accurately recorded, which can be compared with previous studies evaluating gaze behavior during basketball shooting (Vickers, 2007; Vine & Wilson, 2011). We measured the fixation time and fixation point of shooting with eye tracker. The average fixation time told us the average fixation time in a certain area, which can be counted by one or more people. By comparing AOI (the AOI refers to the area in which researchers are interested in stimulus materials), you can determine which areas are actually more important than others. The filter is an IVT filter of fixation. A laptop (ThinkPad E15) installed with ‘Tobii Pro Glasses3’ (Tobii Pro Lab) recording software was incorporated into the system. Tobii Pro lab software provides a complete tool set for performing eye-tracking experiments, from experimental design and data collection to visualization, analysis, and export of eye movement data.

The externally positioned digital camera (HFG50; Canon, Tokyo, Japan) was located 3 m to the right of the participants, perpendicular to their shooting direction (*i.e.*, sagittal plane). This view allowed to capture the entire free throw action of each participant for subsequent offline analysis. Experimenters and devices were located behind participants to minimize interference.

### Time and place

Experiment 1 was conducted on August 22, 2021, and was completed in the basketball hall of Beijing Normal University. The completion of a recording consisted of the following steps: entering the participant name, calibrating the head unit, verifying the calibration (0.5–1 m and 20–40 s, 1 point; as advised by the manufacturer) and starting the recording. Visual information processing and the accompanying calibration procedures had to be newly executed for each participant. Experiment 2 was conducted from August 23, 2021, to October 29, 2021 (excluding National Day) and was completed in the basketball hall of Northwest Normal University. There was a total of 9 weeks of basketball teaching, 90 min of practice time per week and a total of 180 min of practice time. There were 3 basketball shooting tests among them: before the experiment (1st week), during the experiment (5th week) and after the experiment (9th week).

### Statistical analysis

The data were recorded and collected by using Tobii Pro lab software, and the gaze sampling rate of 85% was considered as the standard. SPSS 26.0 (SPSS, Inc., Somers, New York, USA) software was used to calculate the accuracy and conduct statistical analyses. We used the data of FG%, fixation time and fixation point.The AOI time between two groups was compared by using an independent sample t test (Shapiro–Wilk test *P* > 0.05) or Mann–Whitney *U* test (Shapiro–Wilk test *P* ≤ 0.05), and the comparison of FG% and proportion of AOI time was performed by using the Pearson’s chi-squared test. An alpha level of 0.05 was preselected for all of the statistical comparisons. The FG% was calculated by using the number of goals of the total number of shots.

## Results

### Field goal percentage

Pearson’s chi-squared test results of Experiment 1 showed that there was a significant difference in the total FG% between expert players (83.47%) and amateur players (34.86%) (*χ*^2^ = 352.113, *P* = 0.000), and the difference in FG% among the three shooting techniques (free throw, 45° direct shot and 45° bank shot) was significant (*P* < 0.01). The FG% of expert players was significantly higher than that of amateur players. See Table 1.

The Pearson’s chi-squared test results of Experiment 2 showed that there was a significant difference in the total FG% of the three tests between the intervention players (30.19%) and the control players (27.27%) (*χ*^2^ = 4.487, *P* = 0.034). After shooting training, the FG% of the intervention players was significantly higher than that of the control players (*P* < 0.05). There was no significant difference in the total FG% between the intervention players (25.97%) and the control players (25.14%) (*χ*^2^ = 0.131, *P* = 0.717) at the 1st week, and there was no significant difference in the FG% of the three shooting techniques (*P* > 0.05). Before the experiment, the shooting skills of the intervention players and the control players were the same. In addition, there was no significant difference in the total FG% between intervention players (28.19%) and control players (26.53%) (*χ*^2^ = 0.503, *P* = 0.478) at the 5th week, and there was no significant difference in the FG% of the three shooting techniques (*P* > 0.05). Moreover, there was a significant difference in the total FG% of intervention players (36.39%) and control players (30.14%) (*χ*^2^ = 6.335, *P* = 0.012) at the 9th week, and the difference in the FG% of the three shooting techniques was significant (*P* < 0.01). Before the experiment, the FG% of the intervention players and control players was essentially equal. After 5 weeks of practice, there was no significant difference in the FG% between intervention players and control players. After 9 weeks of practice, the FG% of intervention players was significantly higher than that of control players. Furthermore, the training method of the intervention group improved the FG% (see Table 2).

**Table 1:** Comparison of FG% between the expert group and amateur group.

**Shooting techniques**	**Hit (FG%)**		*χ* ^2^	***P*** **value**
	**Expert group**	**Amateur group**		
Free throw	204 (85.00)	101 (42.08)	95.406	0.000^**^
45° direct shot	200 (83.33)	82 (34.17)	119.699	0.000^**^
45° bank shot	197 (82.08)	68 (28.33)	140.196	0.000^**^
Total	601 (83.47)	251 (34.86)	352.113	0.000^**^

**Notes.**

** *P* < 0.01.

**Table 2:** Comparison of FG% between the intervention group and the control group.

**Time**	**Shooting techniques**	**Hit (FG%)**		*χ* ^2^	***P*** **value**
		**Intervention group**	**Control group**		
1st week	free throw	63 (26.25)	60 (25.00)	0.098	0.754
	45° direct shot	65 (27.08)	63 (26.25)	0.043	0.836
	45° bank shot	59 (24.58)	58 (24.17)	0.011	0.915
Total		187 (25.97)	181 (25.14)	0.131	0.717
5th week	free throw	65 (27.08)	63 (26.25)	0.043	0.836
	45° direct shot	70 (29.17)	65 (27.08)	0.258	0.612
	45° bank shot	68 (28.33)	63 (26.25)	0.262	0.608
Total		203 (28.19)	191 (26.53)	0.503	0.478
9th week	free throw	132 (55.00)	70 (29.17)	32.857	0.000^**^
	45° direct shot	130 (54.17)	72 (30.00)	28.754	0.000^**^
	45° bank shot	135 (56.25)	75 (31.25)	30.476	0.000^**^
Total		262 (36.39)	217 (30.14)	6.335	0.012^*^
Total		652 (30.19)	589 (27.27)	4.487	0.034^*^

**Notes.**

* *P* < 0.05.

** *P* < 0.01.

The Pearson’s chi-squared test showed that there was a significant difference in the total FG% of the three tests in the intervention group at the 1st week (25.97%), 5th week (28.19%) and 9th week (36.39%) (*χ*^2^ = 163.900, *P* = 0.000). After shooting training, the FG% of the intervention players significantly increased to a large degree (*P* <0.01). There was no significant difference in the total FG% between the intervention players at the 1st week and the 5th week (*χ*^2^ = 0.900, *P* = 0.343), and there was no significant difference in the FG% of the three shooting total techniques (*P* > 0.05). Additionally, there was a significant difference in the total FG% between the intervention players at the 1st week and the 9th week (*χ*^2^ = 127.032, *P* = 0.000), and the difference in the FG% of the three shooting techniques was extremely significant (*P* < 0.01). There was a significant difference in the total FG% between the 5th week and the 9th week (*χ*^2^ = 107.531, *P* = 0.000), and the difference in FG% among the three shooting techniques was significant (*P* < 0.01). Furthermore, the Pearson’s chi-squared test showed that there was no significant difference in the total FG% of the three tests in the control group at the 1st week (25.14%), 5th week (26.53%) and 9th week (30.14%) (*χ*^2^ = 4.837, *P* = 0.089), and the FG% of the control group was not significantly improved after shooting training (*P* > 0.05). There was no significant difference in the total FG% between the control players in the 1st week and the 5th week (*χ*^2^ = 1.719, *P* = 0.190), and there was no significant difference in the FG% of the three shooting techniques (*P* > 0.05). There was a significant difference in the total FG% between the control players in the 1st week and the 9th week (*χ*^2^ = 4.500, *P* = 0.034), but there was no significant difference in the FG% of the three shooting techniques (*P* >0.05). Moreover, there was no significant difference in the total FG% between the 5th week and the 9th week of the control players (*χ*^2^ = 0.659, *P* = 0.417), and there was no significant difference in the FG% of the three shooting techniques (*P* > 0.05). The training method of the intervention group improved FG% at a faster rate (see Table 3).

### Heatmap

**Figure 1:**
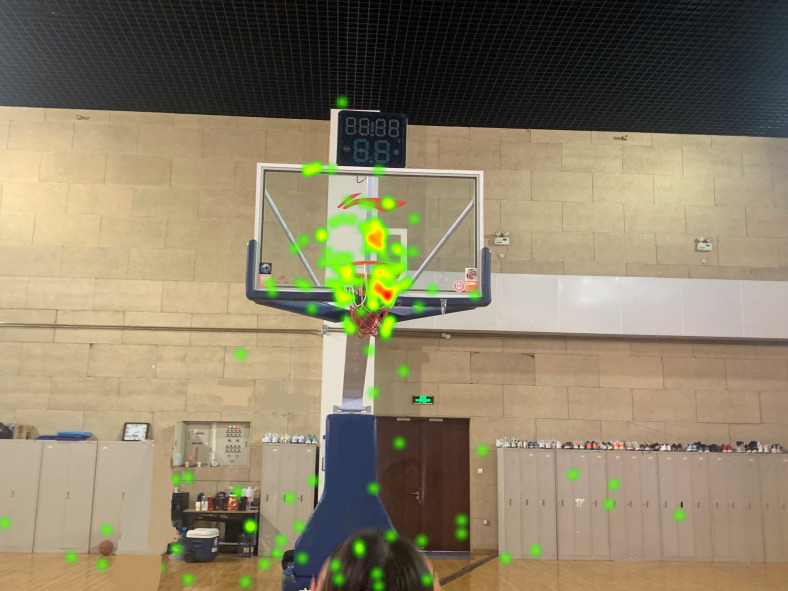
Free throw heatmap of the amateur player. In free throws, the heatmap of the amateur player was mainly localized at the back edge of the hoop.

**Figure 2:**
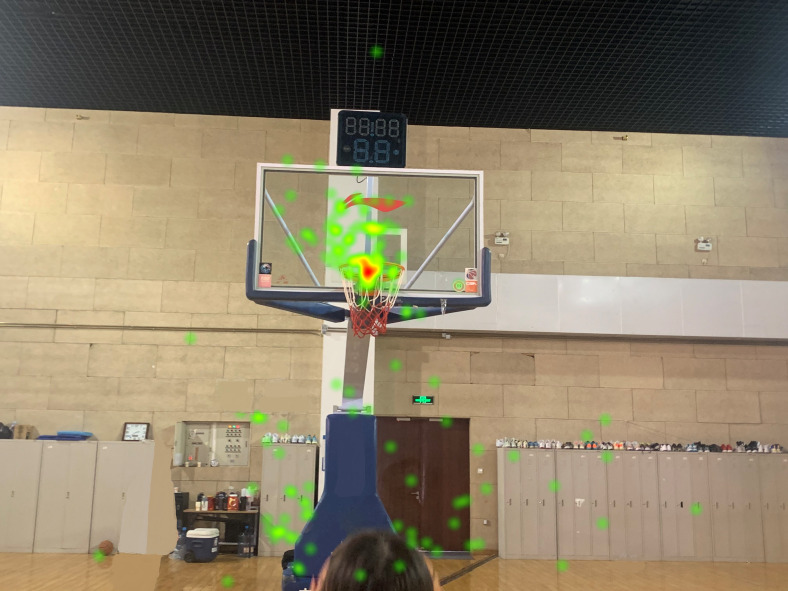
Free throw heatmap of the expert player. In free throws, the heatmap of the expert player was at the front edge of the hoop.

A heatmap included the distribution of the participants’ line of sight measurements on the AOI and was able to visually display the degree of attention of each area by the participants, which can primarily be used for group research (Jin, 2020). In free throws, the heatmap of the amateur group was mainly localized at the back edge of the hoop, whereas the heatmap of the expert group was at the front edge of the hoop. The heatmap of the expert group was more concentrated than that of the amateur group, thus indicating that the expert players have more concentrated visual attention during free throws (see Figs. 1 and 2). This may be one of the reasons why the expert players’ free throw FG% is higher than that of amateur players in this test. These characteristics are the same as for the 45° direct shot and the 45° bank shot.

### AOI time

The purpose of dividing the interest area is to distinguish the gaze index and eye movement data of the participants in each interest area during the experiment. Through interviews with 5 basketball professors, professional athletes and Tobii eye tracker engineers, AOIs were drawn into key interest area-hoops, related interest area-boards and irrelevant interest area-nets (Jin, 2020).

The results of the Mann–Whitney U test showed that there was a significant difference in total shooting time between the expert group (mean: 36.50) and the amateur group (mean: 12.50) (Z = −5.939, *P* = 0.000). The results of the Mann–Whitney U test and independent sample *t* test showed that the time difference between the expert group and the amateur group in the three shooting techniques was extremely significant (*P* < 0.01), and the fixation time difference in each AOI was extremely significant (*P* < 0.01). The shooting action of expert players was significantly faster than that of the amateur players. The results of the Pearson’s chi squared test showed that there was a significant difference between the expert group (43.32%) and the amateur group (19.46%) (*χ*^2^ = 241.418, *P* = 0.000), and there was a significant difference in the proportion of the time of looking at the hoop in the overall shooting time of the three shooting techniques (*P* < 0.01). Moreover, expert players were shown to have longer fixation times at the hoop than amateur players during shooting (see Table 4).

**Table 3:** Intragroup comparison of FG% between the intervention and control groups.

**Time**	**Shooting techniques**	**Intervention group**		**Control group**	
		*χ* ^2^	***P*** **value**	*χ* ^2^	***P*** **value**
1st & 5th week	free throw	0.043	0.836	0.174	0.677
	45° direct shot	0.043	0.836	0.835	0.361
	45° bank shot	0.867	0.352	0.876	0.349
Total		0.900	0.343	1.719	0.190
1st & 9th week	free throw	41.124	0.000^**^	1.055	0.304
	45° direct shot	36.491	0.000^**^	0.835	0.361
	45° bank shot	49.969	0.000^**^	3.006	0.083
Total		127.032	0.000^**^	4.500	0.034^*^
5th & 9th week	free throw	38.649	0.000^**^	0.373	0.542
	45° direct shot	30.857	0.000^**^	0.000	1.000
	45° bank shot	38.319	0.000^**^	0.640	0.424
Total		107.531	0.000^**^	0.659	0.417
Total		163.900	0.000^**^	4.837	0.089

**Notes.**

* *P* < 0.05.

** *P* < 0.01.

**Table 4:** Comparison of AOI time between the expert group and the amateur group.

**Shooting techniques**	**AOI**	**Time** **(M ± SD)**		**Test value**	***P*** **value**
		**Expert group**	**Amateur group**		
Free throw	board	12.50^a^	36.50^a^	5.938^z^	0.000^**^
	hoop	36.50^a^	12.50^a^	−5.939^z^	0.000^**^
	net	12.50^a^	36.50^a^	5.938^z^	0.000^**^
Overall		38.20 ± 1.51	41.58 ± 2.04	−6.539^t^	0.000^**^
Hoop (%)		379.14 (41.33)	146.27 (14.63)	171.220^*χ*^2^^	0.000^**^
45° direct shot	board	7.40 ± 0.79	8.32 ± 0.62	−4.471^t^	0.000^**^
	hoop	36.50^a^	12.50^a^	−5.938^z^	0.000^**^
	net	1.61 ± 0.45	2.65 ± 0.56	−7.046^t^	0.000^**^
Overall		20.17 ± 1.24	16.58 ± 1.14	10.429^t^	0.000^**^
Hoop (%)		269.92 (55.56)	134.42 (33.75)	41.848^*χ*^2^^	0.000^**^
45° bank shot	board	12.50	36.50	5.939^z^	0.000^**^
	hoop	35.08^a^	13.92^a^	−5.238^z^	0.000^**^
	net	12.50^a^	36.50^a^	5.950^z^	0.000^**^
Overall		14.06^a^	34.94^a^	5.259^z^	0.000^**^
Hoop (%)		127.89 (32.74)	80.08 (17.47)	26.587^*χ*^2^^	0.000^**^
Total		36.50^a^	12.50^a^	−5.939^z^	0.000^**^
Hoop (%)		776.95 (43.32)	360.77 (19.46)	241.418^*χ*^2^^	0.000^**^

**Notes.**

a Rank average.

z Mann–Whitney U test.

t Independent sample *t*-test.

*χ* ^2^ Pearson’s chi-squared test.

** *P* < 0.01.

### Participant interviews

In Experiment 1, all participants were interviewed immediately after the experiment. It was found that both the expert and amateur basketball players had specific ‘fixation points’ when shooting. The aiming point of expert players was at the front edge of the hoop, and the aiming point of amateur players was at the center or back edge of the hoop. The interview results were consistent with the results of the eye movement index analysis. Moreover, the participants in the intervention group of Experiment 2 clearly stated that the aiming point was at the front edge of the hoop after 9 weeks of intervention, whereas the control group participants did not change.

## Discussion

The aims of the present study were to examine the differences in the shooting aiming point between expert basketball players and amateur basketball players, as well as the relationship between the shooting aiming point and FG%. There is a large amount of literature on basketball shooting, most of which concerns the kinematics, biomechanics and physics of free throws and jump shots (Miller & Bartlett, 1996). There are some factors that determine the shooting percentage in the identification of the factors, and the effects of variables such as shot height, angle and speed are discussed and studied, sometimes being discussed in conjunction with biomechanical variables such as shoulder angle and torso. Due to the limitations of the instruments, few studies have analyzed visual attention (Yan & Bai, 2018). This study used a wearable eye tracker to investigate the shooting eye movement characteristics of female basketball players with high and low degrees of skill. The results verified our hypothesis. Before a free throw, the basketball player’s relatively sustained attention will enhance performance (Chuang, Huang & Hung, 2013). Moreover, players of the expert group are more accurate when completing technical movements and complete movements at a faster speed (Jin et al., 2020). Our results showed that the total FG% of expert players in the three shooting techniques is more than twice that of amateur players (83.47% *vs.* 34.86%, respectively), and the total time average of expert players is 3 s faster than that of amateur players (74.74 s *vs.* 77.24 s, respectively). The eye movement index results also showed that the expert group players have shorter fixation times, fewer fixations and more reasonable fixation distributions, thus indicating that the expert group players have “efficient” visual attention characteristics in the visual search (Jin, 2020). The exploration of the visual fixation mode of expert players is very important for improving the training efficiency of ordinary players. When most excellent shooters throw hollow balls, they choose the aiming point at the front of the hoop that is closest to the shooting point (Zhang, 2019). Furthermore, amateur players have a wider range of heatmaps than expert players when shooting. In future training, the players should be instructed to have a clear aiming point. When amateur players shoot in the future, the aiming point should be at the front of the hoop (Zhao et al., 2016). This conclusion is different from the finding that the FG% of the aiming of the back hoop is high (Chen & Ren, 2006; Ma, 2002) and aims at the entire hoop when shooting (Mu & Li, 2015). Free throw aiming points are mainly concentrated on the front edge of the hoop, the back edge of the hoop, directly above the centerline of the hoop and somewhere on the centerline of the board, which are mainly used for bank shots (Liu, 2008).

Our aiming point intervention experiment, combined with the participant interviews, fully verified that the shooting aiming point was on the front edge of the hoop. In addition, expert players have a longer fixation time at the hoop during shooting. Long visual fixations are necessary for programming various movement parameters, such as direction, force, velocity, timing and limb coordination (Oliveira, Oudejans & Beek, 2008); performers would use this time for psychological and physiological regulation (Okazaki, Rodacki & Satern, 2015). Furthermore, target fixation duration showed that expert shooters looked at the target area more than twice as long as near experts (972 ms *vs.* 357 ms, respectively) (Oliveira, Oudejans & Beek, 2008), which is the same as our AOI time results. The proportion of the total time of expert players looking at the hoop in the total time of shooting was more than twice that of amateur players (43.32% and 19.46%, respectively). Mechanical analyses of shooting skill, back spin after ball contact with the front hoop and the resultant force make the basketball shooting percentage higher than the forward spin (Zhang, 2014). Coaches can identify the key factors through the visual search of players and report the problems to them in a timely manner (Horn, 2011). Moreover, training on expert wheelchair basketball shooting with a visual constraint forced individuals to use target information as late as possible; after 4 months, training can effectively improve wheelchair basketball shooting (Oudejans et al., 2012). After eight days of quiet eye training, amateur players effectively protected against attentional disruptions associated with performing under pressure (Vine & Wilson, 2011). Three weeks’ Mindfulness training was effectively in improving the levels of mindfulness, attention, and relaxation of elite Chinese shooting athletes (Bu et al., 2019). We observed that the practice time was more than 5 weeks, and aiming at the front hoop can significantly improve the FG% of amateur players, which can effectively guide basketball shooting training.

## Conclusions

Our hypotheses were that the aiming points of expert basketball players and amateur basketball players are different when shooting, which may be the reason for the differences in FG%; additionally, the practice of shooting aiming points can improve the FG%. These findings were supported by the results of our study. Both the expert group and amateur group players had specific aiming points when shooting. Specifically, the expert player aimed at the front of the hoop, whereas the amateur player aimed at the center or back edge of the basket. The front edge hoop aiming points of the expert group were more reasonable. Furthermore, in basketball shooting practice, the practice with an aiming point had a higher FG% than without an aiming point and improved the FG% at a faster rate. Five weeks of training with aiming points did not significantly improve the FG% of amateur players, but 9 weeks of training with aiming points significantly improved the FG%, and the player’s aiming point changed from the center and back edge of the hoop to the front of the hoop. These results demonstrated that the aiming point training can become a new method of shooting improvement in basketball. This study which had certain limitations, and all of the participants’ shooting tests were performed *in situ*. Furthermore, jump shots and mobile shots can be compared in the future.

##  Supplemental Information

10.7717/peerj.14301/supp-1 Supplemental Information 1 Number of goals, AOI time, AOI drawing Shooting skill is typically measured by using field goal percentage , which is the number of shots made out of the total number of shots. AOIs were drawn into key interest area-hoops, related interest area-boards and irrelevant interest area-nets.
The original data shows that expert basketball players have a higher hit rate than amateur basketball players, and their fixation points are more concentrated in the front of the basket frame. The intervention group can effectively improve the shooting percentage after 9 weeks of shooting aiming point practice. But the change of control group was not obvious.

10.7717/peerj.14301/supp-2 Supplemental Information 2 AOI_drawing Free throw, 45° direct shot and 45° bank shot AOI drawing

## Additional Information and Declarations

### Data Availability

The following information was supplied regarding data availability: The raw measurements are available in the Supplementary Files.
