# Supplementary figures and images for: Study on eye movement characteristics and intervention of basketball shooting skill
Source: PeerJ. 2022 Oct 31;10:e14301. doi: 10.7717/peerj.14301 (PMC9632457; doi:10.7717/peerj.14301)

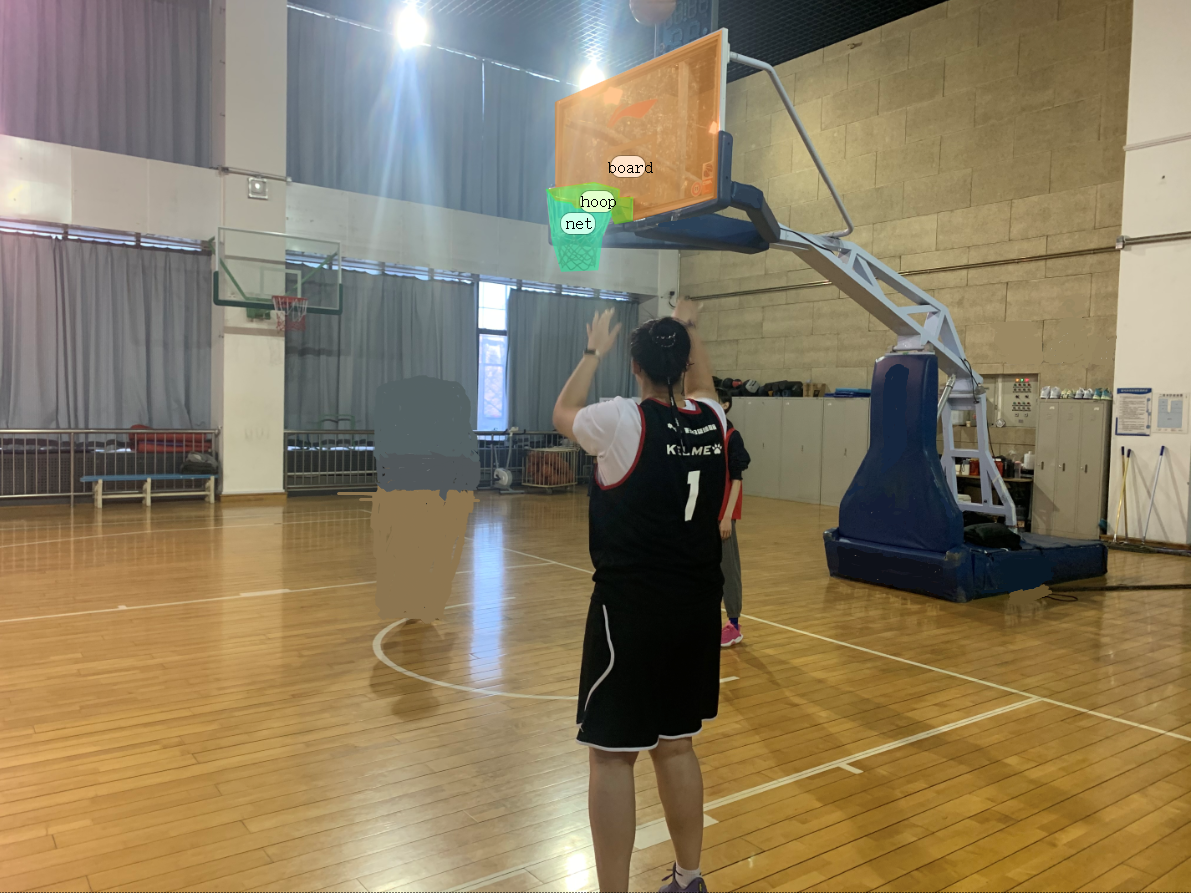

Supplement: Supplemental Information 1 — Shooting skill is typically measured by using field goal percentage , which is the number of shots made out of the total number of shots. AOIs were drawn into key interest area-hoops, related interest area-boards and irrelevant interest area-nets. The original data shows that expert basketball players have a higher hit rate than amateur basketball players, and their fixation points are more concentrated in the front of the basket frame. The intervention group can effectively improve the shooting percentage after 9 weeks of shooting aiming point practice. But the change of control group was not obvious. [file peerj-10-14301-s001.zip › raw data/Bank shot AOI drawing.png]

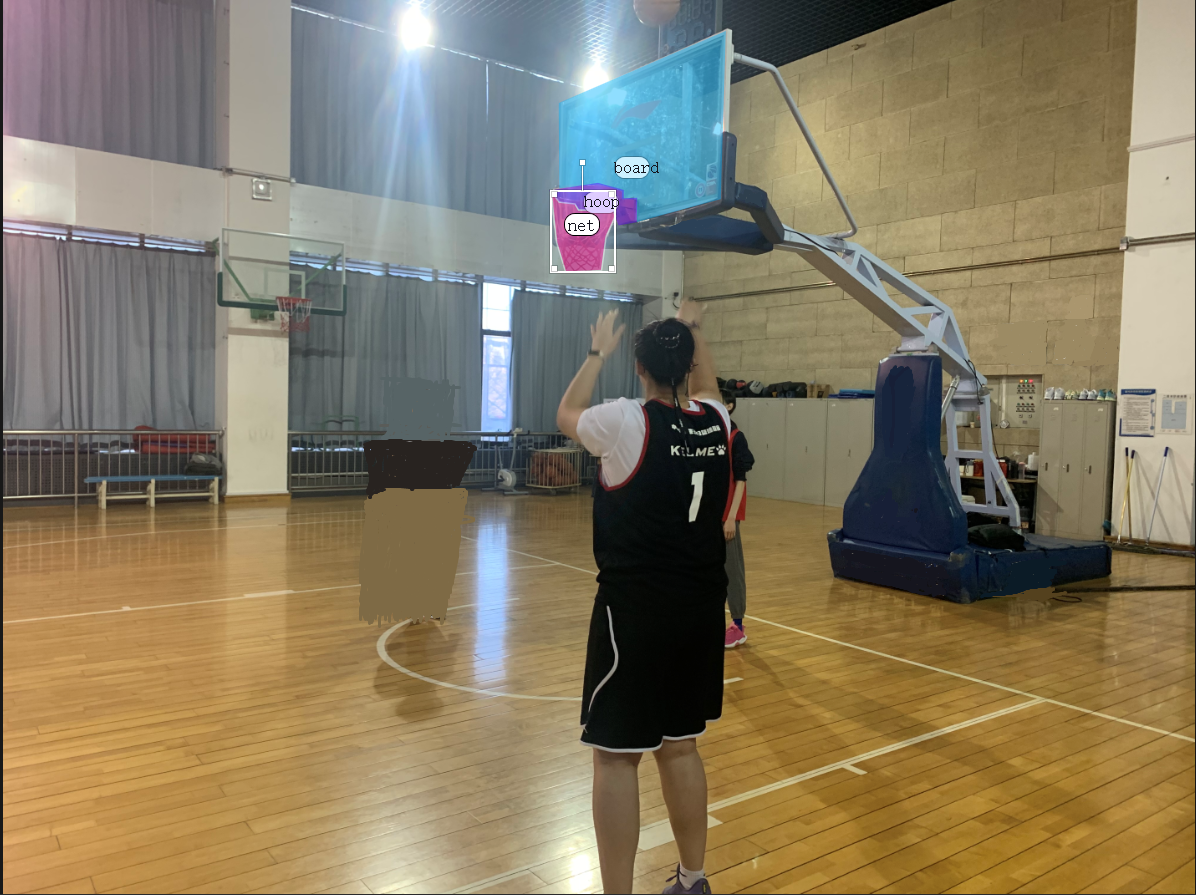

Supplement: Supplemental Information 1 — Shooting skill is typically measured by using field goal percentage , which is the number of shots made out of the total number of shots. AOIs were drawn into key interest area-hoops, related interest area-boards and irrelevant interest area-nets. The original data shows that expert basketball players have a higher hit rate than amateur basketball players, and their fixation points are more concentrated in the front of the basket frame. The intervention group can effectively improve the shooting percentage after 9 weeks of shooting aiming point practice. But the change of control group was not obvious. [file peerj-10-14301-s001.zip › raw data/Direct shot AOI drawing.png]

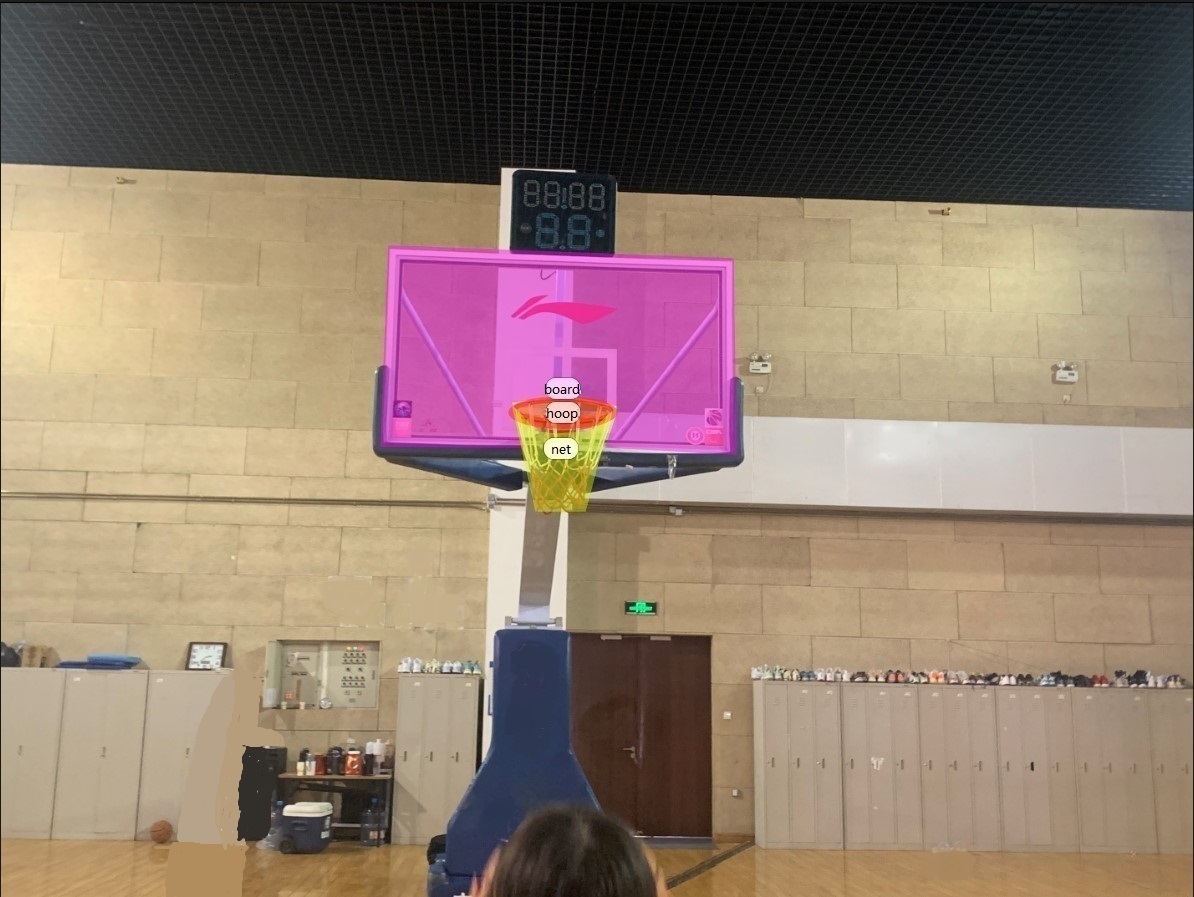

Supplement: Supplemental Information 1 — Shooting skill is typically measured by using field goal percentage , which is the number of shots made out of the total number of shots. AOIs were drawn into key interest area-hoops, related interest area-boards and irrelevant interest area-nets. The original data shows that expert basketball players have a higher hit rate than amateur basketball players, and their fixation points are more concentrated in the front of the basket frame. The intervention group can effectively improve the shooting percentage after 9 weeks of shooting aiming point practice. But the change of control group was not obvious. [file peerj-10-14301-s001.zip › raw data/Free throw AOI drawing.jpg]
